# Dysfunction of Affective Network in Post Ischemic Stroke Depression: A Resting-State Functional Magnetic Resonance Imaging Study

**DOI:** 10.1155/2014/846830

**Published:** 2014-05-14

**Authors:** Peiyao Zhang, Qin Xu, Jianping Dai, Jun Wang, Ning Zhang, Yuejia Luo

**Affiliations:** ^1^Department of Neuroradiology, Beijing Tiantan Hospital, Capital Medical University, No. 6 Tiantanxili, Dongcheng District, Beijing 100050, China; ^2^Centre for Studies of Psychological Application, Key Laboratory of Mental Health and Cognitive Science of Guangdong Province, School of Psychology, South China Normal University, Guangzhou, China; ^3^State Key Laboratory of Cognitive Neuroscience and Learning & IDG/McGovern Institute for Brain Research, Beijing Normal University, 19 Xinjiekouwai Street, Haidian District, Beijing 100875, China; ^4^Center for Collaboration and Innovation in Brain and Learning Sciences, Beijing Normal University, 19 Xinjiekouwai Street, Haidian District, Beijing 100875, China; ^5^Department of Neurology, Beijing Tiantan Hospital, Capital Medical University, No. 6 Tiantanxili, Dongcheng District, Beijing 100050, China

## Abstract

*Objective*. Previous studies have demonstrated that stroke characteristics and social and psychological factors jointly contribute to the development of poststroke depression (PSD). The purpose of this study was to identify altered functional connectivity (FC) of the affective network (AN) in patients with PSD and to explore the correlation between FC and the severity of PSD. *Materials and Methods*. 26 PSD patients, 24 stroke patients without depression, and 24 age-matched normal controls underwent the resting-state functional MRI (fMRI) scanning. The bilateral anterior cingulated cortices (ACCs) were selected as regions of interest (ROIs). FC was calculated and compared among the three groups. The association between FC and Hamilton Depression Rate Scale (HDRS) scores of PSD group was investigated. *Results*. The FC of the AN was disrupted in PSD patients compared to stroke patients without depression and normal controls. Moreover, the left orbital part of inferior frontal gyrus which indicated altered FC was significantly correlated with HDRS scores in PSD patients. *Conclusions*. Dysfunction of the affective network may be one of the reasons of the development of PSD.

## 1. Introduction

Stroke is one of the most common causes of human death [1]. Large-scale stroke survivors suffer from poststroke depression (PSD), which not only hinders recovery but also increases the risk of death significantly [2, 3]. Some studies involving the determinants of PSD have focused on infarction characteristics, including location, size, volume, and white matter lesions, while other studies have proposed that the course of PSD seemed to be dependent on psychosocial factors, such as personality, disability, and social support [4–7]. However, few studies have been conducted to determine the relationship between the risk of PSD and brain lesions or other correlative factors [8–10]. Due to different definitions of depression, the duration of disease, measurement methods, and patient sampling, the pathogenesis of PSD is controversial [6, 9, 10].

In recent years, resting-state functional MRI (fMRI) provided a powerful framework for detecting the mechanism underlying cognitive disorders [11, 12]. The first study of resting-state functional connectivity (FC) showed that spontaneous low-frequency fluctuations in blood oxygen level-dependent (BOLD) signals were exhibited in a sensorimotor network when the brain was in a resting state [13]. FC was considered as the temporal correlation of spontaneous fluctuations in anatomically separated, but functionally related brain regions, and these brain regions were comprised of some specific networks [14]. The most widely explored network is the default mode network (DMN) [15–18].

Moreover, another neural functional network, the affective network (AN), was also detected in a task-related functional MRI study of mood disorders [19–21]. The AN consists of the prefrontal cortex, amygdala, insula, ventral striatum, hippocampus, and anterior cingulate cortex and was associated with emotional activity and modulation [22–25].

We hypothesized that FC in patients with PSD is different from stroke patients without depression and normal controls and the dysfunction of the AN in patients with PSD is associated with the severity of depression. Thus, the objective of this study is to compare FC of the AN among three groups (PSD, stroke without depression, and normal control groups) and to determine the difference between each group. The bilateral anterior cingulate cortices (ACCs) were selected as the region of interest (ROI) in the AN. The correlation between altered FC in PSD patients and severity of depression was also explored.

## 2. Methodology

### 2.1. Subjects

This prospective study was accepted by the local Ethics Committee of Beijing Tiantan Hospital of Capital Medical University. All the subjects signed the written informed consent before participation.

In this study, 50 patients (40–75 years of age) who had their first time ischemic stroke were recruited from the Department of Neurology at Beijing Tiantan Hospital of Capital Medical University between January 2010 and April 2013, including 26 PSD patients (6 females) and 24 stroke patients without depression (5 females). Twenty-four age-matched healthy volunteers were also enrolled as normal control (NC) group (6 females). All the subjects were right-handed.

Patients with PSD were selected consecutively on the basis of the following inclusion criteria: (a) MRI findings of cerebral stroke were confirmed by two senior radiologists; (b) all patients were evaluated by two psychiatrists for depression using the Diagnostic and Statistical Manual of Mental Disorders (DSM-IV, fourth edition); (c) Hamilton Depression Rating Scale (HDRS) 17-item [26] scores >7 were performed to evaluate the depression severity; (d) duration of illness <2 weeks; and (e) patients had experienced their first ischemic stroke and were medication-free.

The inclusion criteria of stroke patients without depression were as follows: (a) diagnosis of stroke performed by two senior radiologists; (b) no depression according to DSM-IV; (c) HDRS 17-item [26] scores <7; (d) duration of illness <2 weeks; and (e) patients having their first ischemic stroke.

Normal controls were referred when they met the following criteria: (a) T2-flair showed no white matter disease; (b) no depression according to DSM-IV; and (c) HDRS scores <7.

Exclusion criteria for all subjects were as follows: (a) other psychiatric diseases, including substance abuse or dependence; (b) other neurological diseases such as dementia; (c) medical disorders impairing cognitive function; (d) a family history of serious psychiatric or neurological illness in first-degree relatives; (e) cerebral hemorrhage or brain trauma; and (f) other contraindications to MR scanning.

### 2.2. MRI Scan Protocol

The MRI scan was performed on a Siemens 3.0T Trio MRI scanner using a standard quadrature head coil. All subjects underwent structural imaging sessions including acquisition of a scout scan with three orthogonal slices, followed by a coarse 3D sagittal T1-weighted magnetization-prepared rapid gradient echo (mp-rage) sequence (176 slices; thickness/gap = 1.0/0 mm; repetition time [TR] = 1900 ms; echo time [TE] = 2.13 ms; flip angle = 9°; matrix 256 × 256; field of view [FOV] = 256 × 256 mm^2^) over the whole brain to automatically compute fMRI slice tilts and offsets that optimize whole-brain coverage parallel to the anterior-posterior commissure plane. The scanning time was 8 minutes. Resting-state functional images were acquired using an echo-planar imaging sequence as follows: TR = 2000 ms; TE = 30 ms; flip angle = 90°; slices = 31; thickness/gap = 3/1 mm; matrix 64 × 64; FOV = 200 × 200 mm^2^. The scanning time was 8 minutes. During the resting-state scanning, all the subjects were instructed to be quiet with their eyes closed and not to think about anything in particular. A sponge mat was used to limit head movement.

### 2.3. Data Analysis

The raw data were preprocessed using SPM8 soft package (http://www.fil.ion.ucl.ac.uk/). With respect to the equilibrium and subject adaptability, the first 10 images of each subject were discarded. Therefore, 230 images for each subject were sequentially put into the following preprocessing procedures. All subjects in this study met the criteria of the head motion less than 3 mm of translation and 3° of rotation in any direction. After motion correction, the images were spatially normalized into standard MNI space and resampled to 3 × 3 × 3 mm^3^, then spatially smoothed with a 4 × 4 × 4 mm^3^ full width at half-maximum Gaussian kernel. After that, a low-pass frequency filter (0.01 < *f* < 0.08 Hz) was applied to reduce physiologic high frequency noise. All the procedures were performed using the REST package (http://www.restfmri.net/forum/).

### 2.4. ROI Selection

**Figure 1:**
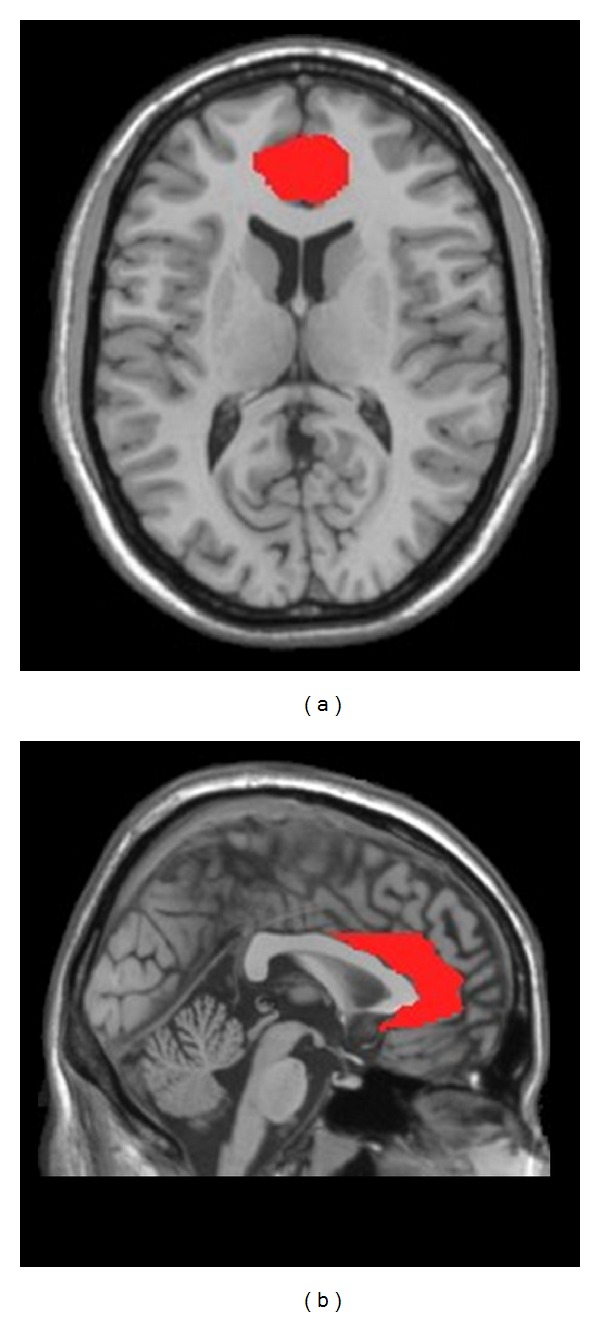
The bilateral ACCs (red region) in axial view (a) and sagital view (b) were extracted from the automated anatomical labeling atlas implemented in MRIcro software. The bilateral ACCs were combined as one-seed region of interest (ROI).

The bilateral ACCs were extracted and combined as one ROI as these areas were defined by previous studies in depressive patients [19, 27]. In the current study, the bilateral ACCs were extracted from the automated anatomical labeling [28] atlas from MRIcro software (http://www.cabiatl.com/mricro/) (Figure 1). Using seed ROI analysis, a seed reference time course was obtained by averaging the time series over the voxels in the bilateral ACCs. Pearson's correlation analysis was carried out between the seed reference and the whole brain in a voxel-wise manner, with the global mean time course, the white matter mean time course, the cerebrospinal fluid mean time course, and the six head motion parameters as nuisance covariates. Then the correlation coefficients were converted to* z*-scores by applying Fisher's* r*-to-*z* transformation {*z* = 0.5ln⁡[(1 + *r*)/(1 − *r*)]}.

### 2.5. Statistical Analysis

In this study, one-way analysis of variance (ANOVA) was carried out to detect comparison of FC across the three groups. Voxels with *P* < 0.01 and cluster size ≥18 voxels were considered significantly different between groups, which corresponds to *P* < 0.01 after correction for multiple comparisons using Monte Carlo simulation (AlphaSim in REST software [http://www.restfmri.net/forum/]). After that, a two-sample post hoc *t* test analyses were performed between each pair of groups (PSD versus stroke, PSD versus NC, and stroke versus NC) to further confirm the between-group differences. Voxels with *P* < 0.01 and cluster size ≥18 voxels were considered significantly different, corresponding to corrected *P* < 0.05 as determined by AlphaSim.

To investigate the relationship between the altered FCs and depression, correlation analyses were performed between the mean* z*-scores of the clusters which showed significant differences among the three groups and the HDRS scores in the PSD group (significant level was *P* ≤ 0.05) using SPSS7 software.

## 3. Result

### 3.1. Subjects

The detailed information of demographic and clinical characteristics of all participants is shown in Table 1. The age, gender, and disease period showed no significant differences among the three groups of subjects.

### 3.2. MRI Image Analysis

By using one-way ANOVA, a significant difference of connection with the seed region was revealed among the three groups.

**Figure 2:**
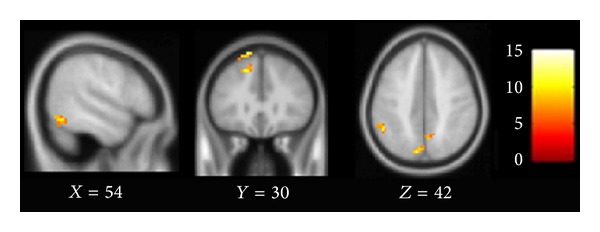
Brain regions which show altered connectivity with ACC among PSD, stroke, and NC groups. Images were thresholded at *F* = 4.91 (*P* < 0.01, corrected by AlphaSim).

**Figure 3:**
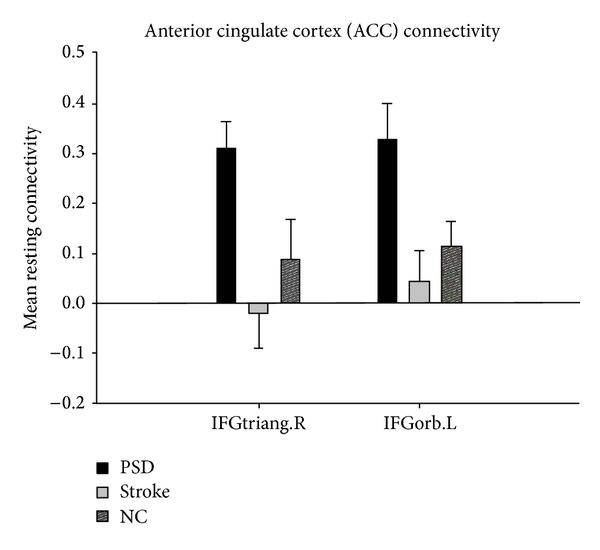
Comparison of PSD, stroke, and NC groups for mean resting connectivity between bilateral ACC and IFGtriang.R and IFGorb.L.

The brain regions which had altered connectivity with ACC in the comparison between the PSD group and two other groups, including the right triangular part of the inferior frontal gyrus (*F* = 8.6, df = 2, and *P* < 0.01) and the left orbital part of inferior frontal gyrus (*F* = 4.01, df = 2, and *P* < 0.01; Figures 2 and 3).

**Figure 4:**
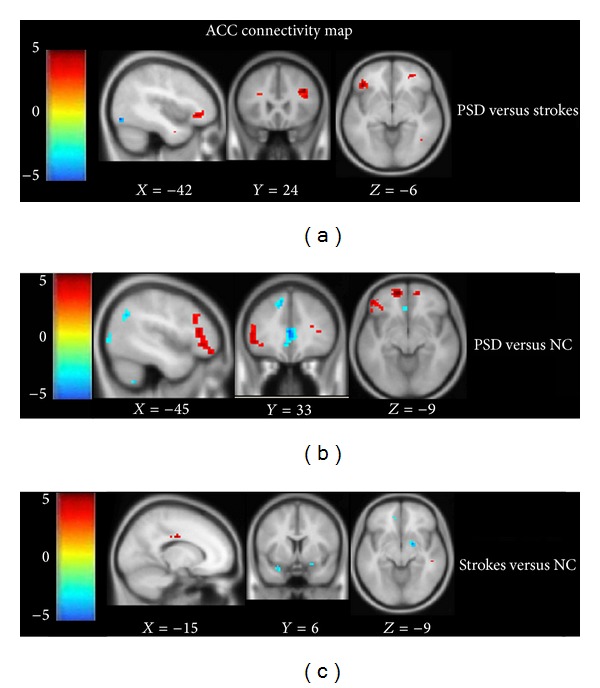
(a)–(c) Comparison of connectivity maps with ACC between two groups. (a) Intergroup difference between PSD and stroke groups; (b) intergroup difference between PSD and NC groups; (c) intergroup difference between stroke and NC groups. The threshold of the images was at *t* = 2.42, *P* < 0.01, corrected by AlphaSim, *P < 0.05.

Further investigation on the detailed differences of FC in the three groups was performed using a* post hoc t* test (*P* < 0.01, cluster size ≥18 was considered significant (Table 2, Figure 4)).

The FC of the left inferior temporal gyrus, left orbital part of the inferior frontal gyrus, and right triangular part of the inferior frontal gyrus was increased with the ACC in the PSD group compared with the stroke group (Figure 4(a)). Furthermore, additional brain regions, including the right inferior temporal gyrus, the left triangular part of the inferior frontal gyrus, the left orbital part of the superior frontal gyrus, and the left middle frontal gyrus had increased FC with the ACC, while the left precuneus and left middle temporal gyrus showed reduced FC with the ACC, existed in the PSD group compared with the NC group (Figure 4(b)). Decreased FC of the left superior temporal gyrus existed in comparison with the stroke and NC groups (Figure 4(c)).

### 3.3. Correlation Analysis

**Figure 5:**
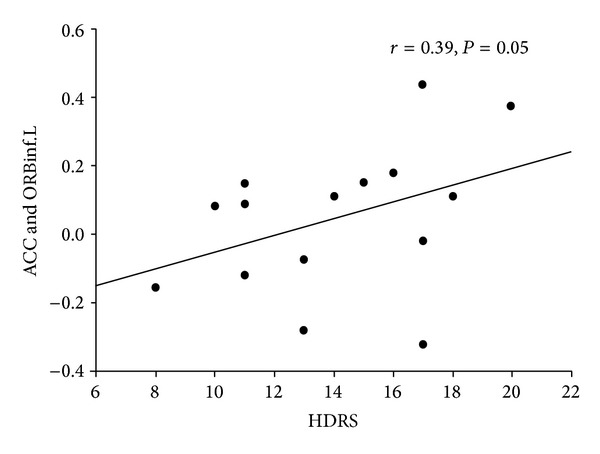
Plots displaying the correlation between the FC (mean* z*-scores) and the HDRS scores in the left orbital part of the inferior frontal gyrus (ORBinf.L) in the PSD patients. HDRS, Hamilton Depression Rating Scale.

Moreover, the FC between the significantly altered clusters (Figure 2) and the seed ROI were computed and then correlated with the HDRS scores of the PSD group (*P* ≤ 0.05). We found that the FC between the left orbital part of the inferior frontal gyrus and ACC was positively correlated with the HDRS scores (*r* = 0.39, *P* = 0.05; Figure 5).

## 4. Discussion

The key finding in this study was that FC of the AN in PSD was altered compared to the stroke and NC groups. Moreover, the altered FC was associated with the HDRS scores in PSD patients. As a result, these findings may support a strong association between the AN impairments and the risk of developing PSD in the subacute phase of stroke.

In the current study, the right inferior temporal gyrus, bilateral triangular part of the inferior frontal gyrus, left orbital part of the inferior frontal gyrus, left orbital part of the superior frontal gyrus, and left middle frontal gyrus showed increased FC with ACC between PSD and NC while the left precuneus and left middle temporal gyrus showed decreased FC with the ACC. The altered regions in PSD compared to stroke were included in the comparison between PSD and NC, except the left inferior temporal gyrus, which showed increased FC with the ACC. Comparing the stroke and NC groups, the left superior temporal gyrus, which showed reduced FC, was absent in the comparison between the PSD and NC. These results suggest that impaired FC of the AN may be related to the development of PSD.

The brain regions which indicated increased FC in the current study were mainly found in the bilateral frontal lobes, which are associated with emotion regulation [29]. The results of the current study are consistent with previous studies, showing that increased FC of right inferior frontal gyrus was found in an affective-related network of depressed patients [30]. Additionally Sheline et al. [27] also suggested that ACC indicated increased FC with bilateral dorsomedial prefrontal cortex. This similarity between PSD and depression may contribute to depressive symptoms which exist both in PSD and depressive patients. A previous study has also reported that the inferior frontal gyrus is implicated in dealing with emotional distraction [31]. Later, another research reported that the inferior frontal gyrus of major depressive patients indicated deactivation in coping with emotional distraction [32]. Accordingly, the increased FC of the bilateral inferior frontal gyrus in the current study may suggest that there is a trend towards inhibition of emotional replication in PSD patients. Furthermore, Cullen et al. [33] found that superior temporal gyrus showed reduced FC with subgenual ACC in adolescence with major depressive disorder. The current study implies a similar finding as decreased FC was shown in the left middle temporal gyrus. Both PSD and depression patients appear to have decreased FC in the temporal lobe, but in different locations. This may suggest that PSD differs from depression. On the other hand, Anand et al. discovered that the FC of bilateral dorsomedial thalamus, amygdala, and left pallidostriatum with ACC was reduced in depressed patients [19]. And the study for patients with bipolar disorder (BDM) and unipolar major depression (MDD) once again proved that depression caused altered FC of bilateral dorsomedial thalamus and amygdala [21]. However, no identical region with them has been found in the present study. The divergence in the present findings from previous studies may be have partly resulted from the difference in illness duration, stages of depressive severity, and the selection of ROI. Nevertheless, to some extent, it may also confirm that PSD is different from depression.

In the current study the correlation between altered FC and HDRS scores has also been detected. The increased FC between the left orbital part of the inferior frontal gyrus and ACC was associated with the severity of depression. Altered FC of the right inferior frontal gyrus, which was considered to be involved in affective processing, has been demonstrated in MDD patients [30]. Zhou et al. [34] suggested that the FC between the left dorsolateral prefrontal cortex and the right middle/superior frontal gyrus was correlated with the HDRS score. Another study suggested that there was an association between left frontal impairment and the severity of depression in affective PSD patients [35]. These results were consistent with our findings and may confirm the speculation that FC impairment of the AN was associated with PSD. However, although the association between frontal FC damage and severity of depression was indicated both in previous studies and in the current study, the specific location was different. Moreover, there was a negative correlation between the left hippocampus and HDRS scores, while the positive correlation was found between the left caudate nucleus and HDRS scores [36]. These differences may be due to the differences in samples, selection of ROI, and depression severity but may also confirm that PSD is different from depression.

The limitations of this study were that the lesion location, volume and correlation with the altered FC were not considered. These additional studies such as comparing the lesion characteristics between the PSD and stroke patients without depression groups will be performed in the future studies. In the previous study, the central coordinates of ROIs were based on the task-related state fMRI study for depressive patients [28]. In the present study, a task-related state fMRI study was not performed because of the subacute phase of the stroke patients.

## 5. Conclusion

In this study, altered FC of brain regions which belongs to the AN has been found in PSD patients, and significant differences also have been found between PSD and stroke without depression patients according to the AN. Furthermore, altered FC is correlated with HDRS scores in PSD patients. From these findings we can infer that the dysfunction of the AN may be one of the causes of PSD.

## Figures and Tables

**Table 1:** Demographic and clinical characteristic of the samples.

	PSD (*n* = 26)	Stroke (*n* = 24)	NC (*n* = 24)
Age	56.4 ± 10.2	60.2 ± 9.8	57.1 ± 9.5
Gender			
Male	20	19	18
Female	6	5	6
HDRS score	12.5 ± 3.87	—	—
Disease period (day)	9.92 ± 2.77	9.62 ± 2.72	—

HDRS: Hamilton Depression Rating Scale. Unless otherwise indicated, data are expressed as the mean ± SD. NC: normal control.

**Table 2:** Brain regions show different FC with ACC between two groups.

Regions	BA	Cluster size	Maximal *t*-score	Primary peak location		
				(MNI)		
PSD versus Stroke						
Positive						
ITG.L	21	33	3.27	−45	3	−36
ORBinf.L		35	4.01	−42	30	−6
IFGtriang.R		21	4.18	36	24	26
Negative	none	none	none	none	none	none
PSD versus NC						
Positive						
ITG.R	37	42	3.15	51	−63	−15
IFGtriang.L		105	4.15	−48	27	12
ORBinf.L		66	4.01	−42	30	−6
ORBsup.L		32	4.12	−12	57	−9
MFG.L	10	66	3.92	−39	48	6
IFGtriang.R		34	3.64	36	24	26
Negative						
PCUN.L	7	30	−3.03	−9	−61	34
MTG.L		22	−3.84	−57	−63	3
Strokes versus NC						
Negative						
TPOsup.L		18	−3.12	−30	6	−24

Significant level of *P* < 0.01, cluster size ≥ 18 with AlphaSim correction for multiple comparisons, *P < 0.05; NC: normal control; BA: Broadman area; MNI: Montreal Neurological Institute spatial array coordinates; ITG: inferior temporal gyrus; ORBinf.: inferior frontal gyrus, orbital part; IFGtriang.: inferior frontal gyrus, triangular part; ORBsup.: superior frontal gyrus, orbital part; STG: superior temporal gyrus; MFG: middle frontal gyrus; ANG: angular gyrus; SFG: superior frontal gyrus; PCUN: precuneus; MTG: middle temporal gyrus; TPOsup.: superior temporal gyrus; L: left; R: right.
